# Path from discovery to recovery: therapeutic and diagnostic advances in Alzheimer's dementia

**DOI:** 10.1007/s00415-023-11840-w

**Published:** 2023-07-03

**Authors:** Aung M. Saw, James Hrastelj, Neil P. Robertson

**Affiliations:** 1 Department of Neurology, University Hospital of Wales, Heath Park, Cardiff, CF14 4XN UK; 2 Institute of Psychological Medicine and Clinical Neuroscience, Cardiff University, University Hospital of Wales, Heath Park, Cardiff, CF14 4XN UK

## Introduction

Alzheimer's Dementia (AD) is a progressive neurodegenerative disorder characterized by cumulative memory loss, cognitive decline and maladaptive behaviour. Despite being a leading cause of death, it has no currently widely available treatment to prevent, slow or stop disease progression. It has been estimated that the number of people expected to die from AD is predicted to quadruple by 2040 unless there are advances in clinically meaningful diagnostic and therapeutic discoveries.

An initial theory on the underlying pathogenesis of AD was proposed by Alois Alzheimer in 1906, and is known as “the amyloid cascade hypothesis”. It proposes that beta-amyloid plaques outside of neurons not only act as neurotoxins but also trigger abnormal phosphorylation and aggregation of tau proteins, which form neurofibrillary tangles inside neurons. The combined effect of those two deposits ultimately leads to neurodegeneration, synaptic dysfunction, and cognitive impairment. Over the last 3 decades, researchers have focussed on pharmacological therapies that target beta-amyloid plaques and tau protein, albeit without much success. Developing a clinically meaningful therapy has been challenging for several reasons including; flawed timing of intervention in the AD Continuum, inadequate target engagement within the blood–brain barrier, the complexity of the underlying pathogenesis, lack of universally acceptable study design, patient selection and safety concerns. However, despite these delays and difficulties, the US Food and Drug Administration (FDA) has recently approved Aducanumab, the first disease-modifying therapy for AD.

In this month's journal club, we review two recent clinical trials that target beta-amyloid plaques and Tau protein in early AD. In addition, we discuss a report of novel imaging methodology that may allow the identification of processes that precede neuronal death and therefore offer possibilities for very early diagnosis.

## Lecanemab in early Alzheimer's disease

The Clarity AD trial was a multi-centre, double-blinded, phase 3, randomized controlled trial conducted in 1795 people with early Alzheimer's disease to determine the effectiveness and safety profile of Lecanemab (Humanized IgG1 monoclonal antibody to amyloid-beta soluble protofibrils). Participants were randomly assigned to receive either Lecanemab (10 mg/kg of body weight) or placebo every 2 weeks for 18 months in a 1:1 ratio. The primary endpoint was a change from baseline at 18 months in Clinical Dementia Rating–Sum of Boxes (CDR-SB) score (range 0–18 with higher scores indicating greater impairment). Participants were aged 50–90 with similar baseline attributes, randomised according to the clinical subgroup (mild cognitive impairment due to AD or mild AD), the presence or absence of concomitant symptomatic medications for AD, Apolipoprotein E4 carriers or non-carriers and geographic location. Care was taken to include participants from underrepresented ethical groups, but the majority of the participants were Caucasian (76.85%).

Results at 18 months demonstrated a mean change in CDR-SB score of 1.21 in the Lecanemab group in comparison to 1.66 in the placebo group (difference − 0.45, 95% confidence interval, *p* < 0.001). The Lecanemab group also displayed a slower decline in other measures of cognitive function, including ADAS-cog14 score (Alzheimer’s Disease Assessment Scale), ADCOMS (Alzheimer’s Disease Composite Score) and ADCS-MCI-ADL(Alzheimer’s Disease Cooperative Study- Activities of Daily Living Scale for Mild Cognitive Impairment) score. In addition, results of analyses of Cerebrospinal fluid (CSF) and plasma biomarkers and amyloid burden on Positron Emission Tomography (PET) scan in pre-specified subgroups revealed improvement for all assessments in the Lecanemab group (with the exception of CSF Neurofilament Light). Adverse events in Lecanemab groups included infusion-related reactions (26.4%) and Amyloid Related Imaging Abnormalities (ARIA) with oedema or effusions (ARIA-E) (12.6%).

*Comment*: lecanemab represents a significant advance in the development of anti-amyloid therapies in AD. More studies are required to demonstrate equivalent efficacy in underrepresented ethic groups such as Black and Hispanic populations. In addition, some practical issues need to be addressed including the significant dropout rate (during the COVID pandemic) and the potential impracticality of long-term monitoring with neuroimaging for ARIA in a real-world setting, particularly in developing countries. Further trials are needed to assess the efficacy and safety of Lecanemab over a longer follow-up period and within wider and more diverse populations.*van Dyck CH et al. N Engl J Med. 2023 Jan 5;388(1):9–21.*

## Tau-Targeting antisense oligonucleotide MAPTrx in mild Alzheimer’s disease: a phase 1b, randomized, placebo-controlled trial

Phosphorylated Tau is considered a key driver of neurodegeneration in AD and is encoded by the microtubule-associated protein tau (MAPT) gene. MAPTrx (ISIS 814907/BIIB080) is an antisense oligonucleotide (ASO) gene therapy, designed to reduce concentrations of MAPT messenger RNA when administered intrathecally. Mummery et al. conducted a randomized, double-blinded, placebo-controlled, multiple-ascending dose phase 1b trial to evaluate the safety (primary outcome), pharmacokinetics, and target engagement (secondary outcomes) of MAPTrx in 46 patients with mild Alzheimer's Disease (AD). Participants were enrolled into four ascending dose cohorts and randomized 3:1 to intrathecal bolus administration of MAPTrx (10 mg,30 mg, 60 mg or 2 quarterly doses at 115 mg) or placebo every 4–12 weeks during a 13-week treatment period, during which biomarker and neuroimaging monitoring was also undertaken.

The primary result of the trial showed that MAPTrx is well tolerated with no serious adverse effects related to the study medication. The commonest reported adverse event in the MAPTrx group was a post-lumbar puncture (LP) headache, which was generally considered to be mild with no participant requiring a blood patch. There were no reports of suicidal behaviour or serious suicidal ideation in any of the participants. Secondary outcome analysis revealed that the median peak plasma concentrations of the study medicine were achieved within 4 h of intrathecal administration and declined to less than 30% by 24 h after administration. There was a dose-dependent reduction in CSF tau levels in the MAPTrx group, most pronounced in participants receiving a dose of 60 mg, of up to 49%.

*Comment*: this first-in-human study has demonstrated that MAPTrx engaged its target, as evidenced by the marked dose-dependent and sustained reductions in CSF t-tau concentration, and has an acceptable initial safety profile. Limitations, as always in these early studies, include the small cohort size. In addition, all participants were Caucasians and had a relatively young median age of 66 years. Clearly, the next steps will need to engage larger trials encompassing greater ethnic and age diversity as well as exploring the longitudinal trajectory of the clinical and pharmacodynamic effect of MAPTrx whilst observing longer-term safety data.

*Mummery CJ et al. Nat Med. 2023 Apr 24.* https://doi.org/10.1038/s41591-023-02326-3 *. Epub ahead of print. PMID* *:* *37,095,250.*

## Visualizing reactive astrocyte-neuron interaction in Alzheimer's disease using 11C-acetate and 18F-FDG

Astrocytes are glial cells in the brain that can display dramatic morphological and functional changes when exposed to various physical and chemical insults. Reactive astrogliosis was long thought to be a hallmark of neuroinflammation in AD. However, there is no currently clinically validated neuroimaging technique available to visualise this process. In this study, the authors first demonstrated that reactive astrocytes in the AD brain (of rodent and post-mortem human) exhibit elevated acetate uptake through increased expression of monocarboxylate transporter 1 (MCT1). They were then able to show that increased acetate uptake led to the release of the inhibitory neurotransmitter, Gama-aminobutyric Acid (GABA), which subsequently hindered glucose metabolism by reduced expression of Glucose Transporter 3 (GLUT3). They then used astrocyte-specific energy substrate11C-acetate, to augment traditional PET scans to visualize the elevated acetate uptake in reactive astrocytes and then used 18F-Fludrodeoxyglucose (FDG) deployed PET scan to picture associated neuronal glucose hypometabolism.

Proof that the two mechanisms were dependent on each other was provided by using the same imaging method which verified that brain areas demonstrating a significant increase in 11C-acetate uptake were identical to those which showed a significant reduction in F-FDG uptake. Finally, a proof of concept human study was performed in 11 AD patients and 10 healthy controlled subjects where they found a strong correlation between patients' cognitive function and the PET signals of both 11C-acetate and 18F-FDG, validated neurophysiologically by the Seoul Neurophysiological Screening Battery (SNSB) score and clinically by Mini-Mental State Examination (MMSE) score.

*Comment*: this study demonstrates that PET imaging when combined with 11C-acetate and F-FDG can visualize the reactive astrocyte-neuron interaction in AD patients. It is particularly useful due to two distinct safety benefits of 11C-acetate in clinical use including being an abundantly ubiquitous molecule in vivo and having a short half-life. It also highlights potential targets for future pharmacological therapies such as MCT1 and GLU3 transporters. However, this was a small single-centre unblinded study and follow-up studies with larger sample size and robust study design are needed to validate and explore the generalisability of these initial findings.

*Min-Ho N et al. Brain. 2023 Apr 17:awad037. *https://doi.org/10.1093/brain/awad037*. Epub ahead of print. PMID: 37,062,541.*

